# Impacts of Senolytic Phytochemicals on Gut Microbiota: A Comprehensive Review

**DOI:** 10.4014/jmb.2408.08032

**Published:** 2024-11-18

**Authors:** Hee Soo Kim, Chang Hwa Jung

**Affiliations:** 1 Aging and Metabolism Research Group, Korea Food Research Institute, Wanju-gun, Jeollabuk-do 55365, Republic of Korea; 2 Department of Food Biotechnology, University of Science and Technology, Wanju-gun, Jeollabuk-do 55365, Republic of Korea

**Keywords:** Senolytic, senescence, aging, gut, phytochemical

## Abstract

There is increasing interest in utilizing senolytics to selectively remove senescent cells from intestinal tissues, with the aim of maintaining a healthy gut environment during aging. This strategy underscores the potential of senolytics to enhance gut health by delaying intestinal aging and positively modulating gut microbiota. Certain plant-based phytochemicals have demonstrated promising senolytic effects. Beyond their ability to eliminate senescent cells, these compounds also exhibit antioxidant and anti-inflammatory properties, reducing oxidative stress and inflammation-key drivers of age-related diseases. By selectively removing senescent cells from the intestine, senolytic phytochemicals contribute to an improved intestinal inflammatory environment and promote the growth of a diverse microbial community. Ultimately, the dietary intake of these senolytic phytochemicals aids in maintaining a healthier intestinal microenvironment by targeting and clearing aged enterocytes.

## Introduction

Aging cells secrete a complex mixture, known as the senescence-associated secretory phenotype (SASP), with high levels of inflammatory cytokines, chemokines, growth factors, and proteases [1]. These factors induce senescence in the surrounding healthy cells [2]. Therefore, inhibiting the accumulation of senescent cells is crucial for healthy aging and the prevention of various age-related diseases. Senolytics, derived from the word “senescence” and “lytic” (to break down), are a novel class of drugs designed to target and eliminate senescent cells, thereby delaying aging. Senolytics suppress SASP expression by selectively removing senescent cells and retaining healthy cells [3].

Cellular senescence is a stable and irreversible form of cell cycle arrest that occurs in response to various intrinsic and extrinsic stimuli, such as oncogene activation, oxidative stress, mitochondrial dysfunction, irradiation, and chemotherapy [4-8]. Senescent cells activate a “pro-survival network” to resist apoptosis, and senolytics induce cell death by disrupting this network [9]. U.S. Food and Drug Administration-approved drugs, such as dasatinib and quercetin, have been reported as promising senolytic candidates [10]. Quercetin, a flavonoid found in onions, demonstrates the potential use of phytochemicals as senolytics. The beneficial use of this chemical has increased interest in the identification of other natural phytochemicals with senolytic properties and minimal side effects.

## Targeting Key Survival Pathways of Senescent Cells

Senolytic development mainly focuses on neutralizing the key pro-survival pathways in senescent cells. These targeted pathways include anti-apoptotic pathways involving mitochondrial apoptosis, cell cycle arrest, signal transduction, and stress-response pathways [11-14]. A key mechanism by which senolytics exert their effects is through the induction of mitochondrial apoptosis by targeting B-cell lymphoma 2 (Bcl-2) and Bcl-extra-large (xL) [15]. Fisetin, a plant-derived flavonol, inhibits anti-apoptotic proteins and facilitates the activation of pro-apoptotic factors, such as Bcl-2-associated X (Bax) and Bcl-associated killer (Bak) [16]. This process leads to permeabilization of the outer mitochondrial membrane and subsequent release of cytochrome C, thereby inducing apoptosis in senescent cells. Additionally, tumor suppressor protein p53 is a major target of senolytics. p53 plays a crucial role in maintaining cellular homeostasis by inducing cell cycle arrest and promoting apoptosis, thereby eliminating the damaged and potentially cancerous cells [11]. Enhancing the activity of p53 using murine double minute 2 antagonist nutlin-3a induces apoptosis in senescent cells [17]. Another p53-targeting strategy involves disrupting the interaction between forkhead box (FOX)-O4 and p53. FOXO4-D-retro-inverso (FOXO4-DRI) is a synthetic peptide engineered to interfere with this interaction, which is crucial for the survival of senescent cells. By competitively inhibiting the binding of FOXO4 to p53, FOXO4-DRI releases p53 and activates the apoptotic pathway [18].

Dysregulation of signal transduction pathways often contributes to the survival of senescent cells. Inhibition of the phosphatidylinositol 3-kinase (PI3K)/AKT pathway suppresses cell survival and proliferation, leading to increased apoptosis in senescent cells [19]. This inhibition also affects other pathways, enhancing the overall senolytic effects. Src family kinases, including Src, Fyn, and Yes, play crucial roles in various cellular processes, such as cell proliferation, differentiation, and survival [20]. Inhibition of Src kinases effectively eliminates senescent cells by disrupting these pathways [21]. Src kinase inhibitors can be used in immunotherapy and geriatric care to target cancer cells and treat age-related chronic inflammatory diseases [22]. Heat shock protein 90 (HSP90), a molecular chaperone managing cellular stress, plays crucial roles in the maintenance and survival of senescent cells [23]. Targeting HSP90 in senescent cells can disrupt critical survival pathways leading to increased apoptosis. Various inhibitors, such as geldanamycin, 17-AAG, and 17-DMAG selectively reduce the viability of senescent cells by targeting HSP90 [14]. This strategy exploits the excessive stress-dependence of senescent cells over normal cells.

**Fig. 1:**
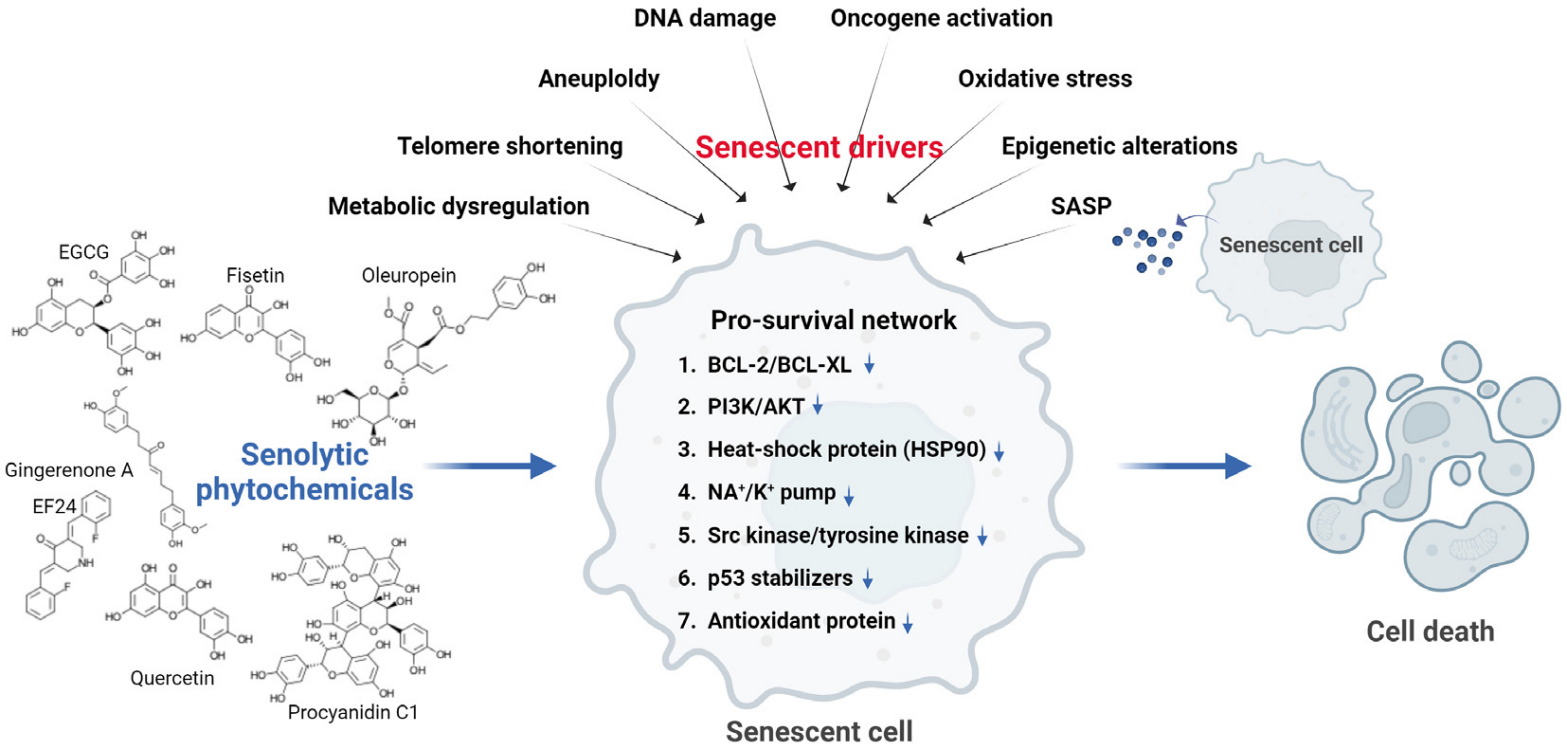
Molecular markers and pathways involved in senescent cell death.

In addition to these mechanisms, cardiac glycosides affect sodium-potassium adenosine triphosphatase (Na^+^/K^+^-ATPase) transport enzymes in the cardiovascular and renal tissues. This induces cell death by causing an imbalance in the intracellular electrochemical gradient, leading to depolarization and acidification [24]. Oxidation resistance 1 (OXR1) is an antioxidant protein that plays a crucial role in cellular defense against oxidative stress [25]. It enhances the antioxidant capacity of cells by regulating the expression of various antioxidant enzymes essential for maintaining redox homeostasis and protecting the cells from oxidative damage. Inhibiting the function of OXR1 eliminates these antioxidant defense mechanisms and increases reactive oxygen species (ROS) generation, resulting in cell death [26]. Senescent cells also exhibit altered autophagic activity. Modulation of autophagy using compounds, such as rapamycin and caloric restriction mimetics, affects the survival of senescent cells [27, 28]. Although these compounds are not classical senolytics, they modulate SASP and contribute to the reduction of senescent cell burden. Representative molecular targets of senolytics are shown in Fig. 1.

## Phytochemicals with Senolytic Effects

Phytochemicals with senolytic effects can induce senescent cell death, thereby reducing inflammation derived from senescent cells and improving tissue function. However, among the various phytochemicals, only a few exert senolytic effects. Notable phytochemicals with senolytic effects include quercetin, fisetin, and curcumin [29-31]. Quercetin was the first reported senolytic phytochemical, targeting multiple pathways including the PI3K/AKT and BCL-2 pathways, and reducing senescence in human umbilical vein endothelial cells (HUVECs) and mouse embryonic fibroblasts (MEFs) [13, 29]. Combined with dasatinib, quercetin effectively reduces the number of senescent cells in various tissues, improves the physical functions and cognitive abilities, and extends the lifespan in animal models [32]. In the first human trial, this combination effectively reduced the aging-related marker levels in the blood, skin, and fat tissues [10] and improved the physical functions in a small population of patients with idiopathic pulmonary fibrosis [33]. Fisetin also targets several signaling pathways, including the PI3K/AKT and BCL-2 pathways, decreasing the survival of senescent cells by inhibiting these pathways [30]. Fisetin reduces the number of senescent cells, alleviates inflammation, and improves cognitive function and physical performance in aging mice [34]. Curcumin exhibits weak senolytic activity in primary MEFs from Errc1^-/-^ mice [30]. EF24, a curcumin analog with senolytic properties, induces senescent cell death by reducing BCL-2 family protein levels in human WI-38 fibroblasts, IMR-90 fibroblasts, HUVECs, and human renal epithelial cells [35]. Epigallocatechin gallate (EGCG), a representative functional phytochemical found in green tea, exhibits senolytic activity by inducing the p53-mediated cell cycle arrest and inhibiting the PI3K/AKT and BCL-2 pathways in 3T3-L1 preadipocytes, whose senescence is induced by H_2_O_2_ [36].

Polyphenol procyanidin C1 improves the lifespan and life expectancy in mice through senolytic effects [37]. It selectively eliminates senescent cells by promoting ROS production and inducing mitochondrial dysfunction. This effect is associated with increased NADPH oxidase activator, p53-upregulated modulator of apoptosis, and increased cleaved caspase-3 levels. Gingerenone A, a polyphenol found in ginger that is structurally similar to curcumin, induces apoptosis by reducing the expression of the anti-apoptotic protein, Bcl-xL, in aged WI-38 human fibroblasts induced by ionizing radiation [38]. Oleuropein, the most abundant polyphenol in olive leaves and fruits, inhibits the accumulation of senescent cells in primary cultured osteoarthritic chondrocytes [39]. Oleuropein treatment significantly reduces the number of accumulated senescent cells and decreases the levels of aging biomarkers, p16^Ink4a^ and p53/p21^Cip1^. It also attenuates the secretion of SASP factors, such as interleukin (IL)-6, cyclooxygenase-2, and IL-1β. Phytochemicals with senolytic effects described above are listed in Table 1.

## Senolytic Phytochemicals Improve Microbial Dysbiosis

**Fig. 2:**
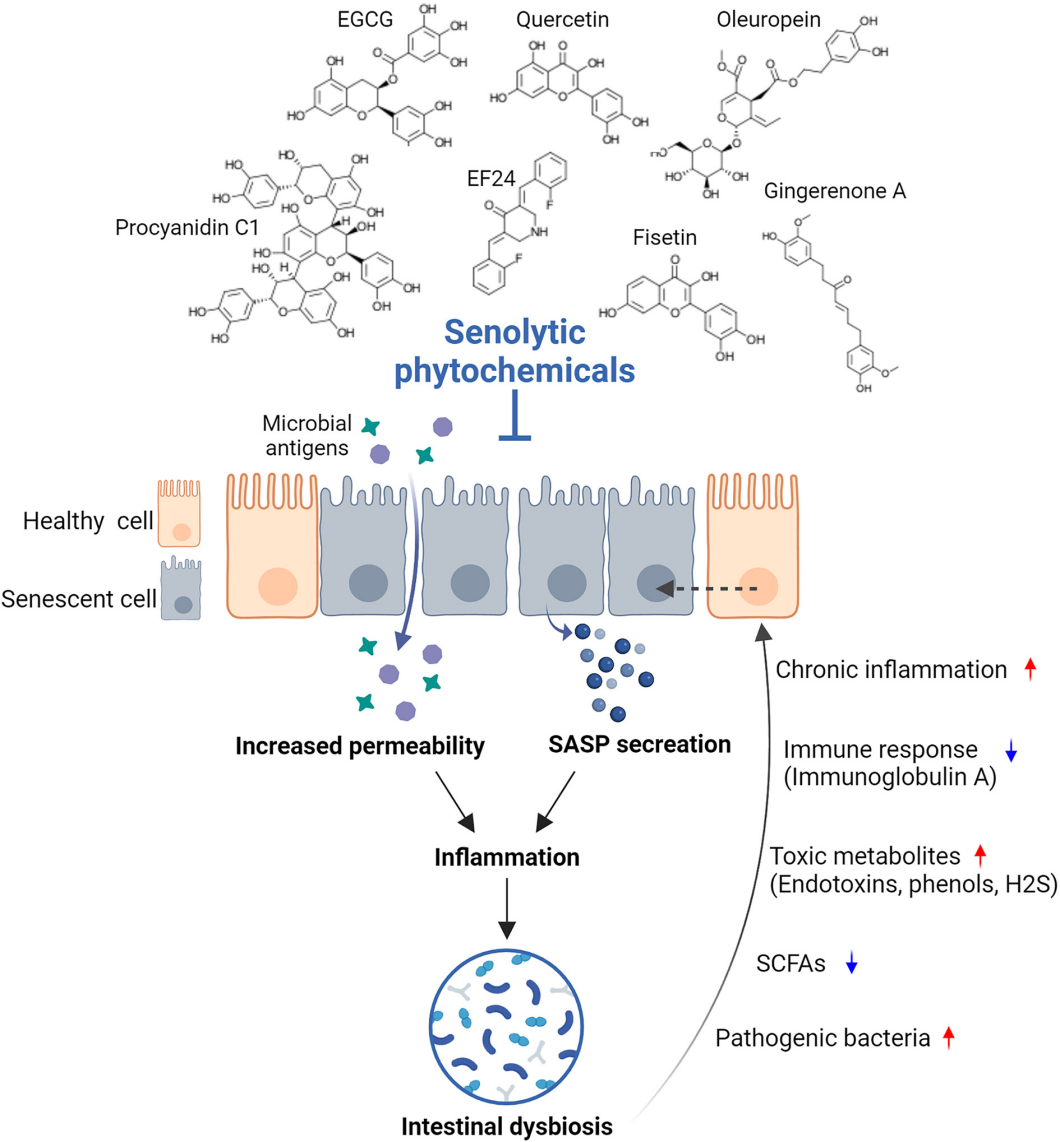
Schematic diagram illustrating the effects of senolytics on aged intestinal cells.

The intestinal mucosal tissue harbors the largest number of symbiotic microorganisms in the human body and plays a crucial role in maintaining homeostasis between the host and the gut microbiota by suppressing unnecessary immune responses to harmless antigens and cooperating with immune cells [40]. However, as intestinal cells age, SASP factor levels increase, accelerating the aging of surrounding cells and increasing intestinal inflammation [41]. Aged enterocytes also contribute to an imbalance in intestinal microbiota [42]. Age-related microbial dysbiosis can cause enterocyte aging by altering metabolites and immune responses, resulting in chronic inflammation (Fig. 2) [43, 44]. Combination of senolytic dasatinib and quercetin not only mitigates intestinal aging but also improves microbial imbalance associated with aging by enhancing the immune function [45]. Senolytics treatment decreases markers of senescent cells (*p16^Ink4a^* and *p21^Cip1^*) and markers of SASP and inflammation (*Cxcl1*, *Il1β*, *Il6*, *Mcp1*, and *Tnfα*) in the ileum, cecum, and colon, indicating that dasatinib and quercetin reduce intestinal cellular senescence and the inflammation burden in aged mice. Additionally, senolytic treatment decreases the Firmicutes/Bacteroidetes ratio and increases the abundance of *Akkermansia muciniphila*, which is negatively correlated with intestinal inflammation and aging. Moreover, senolytic treatment increases the abundance of other taxa, including *Ruminococcus*, *Dorea*, *Coprococcus*, *Oscillospira*, *Bacteroides*, and *Sutterella*. Most of these taxa are negatively correlated with various inflammatory and aging markers, suggesting that senolytic treatments beneficially modulate the gut microbiota. In an inflammatory bowel disease (IBD) model, where the accumulation of senescent cells leads to the release of SASP factors that propagate inflammation, fisetin treatment inhibits expressions of *p53*, *p16*, *Bcl2*, *Cxcl1*, *Mcp1*, and miRNAs related to senescence and inflammation. Fisetin also improves gut health by increasing the beneficial gut bacterium *Akkermansia muciniphila* and improving microbial imbalance [46]. This suggests that senolytic treatment can mitigate the pro-inflammatory environment by reducing the senescent burden and down-regulating SASP factors, which may support the maintenance of beneficial gut bacterium. Studies have revealed specific and distinct gut microbiome signatures associated with senolytic treatment that are directly or indirectly linked to changes in enterocyte senescence and the inflammatory state in aged mice, while the intervention did not alter the microbial composition in young mice. However, further studies are necessary to assess whether the interactions between senolytics and intestinal microbiota are direct or mediated via altered host physiology and metabolism.

Although research on the mechanisms by which senolytic phytochemicals regulate aged intestinal cells and improve the intestinal environment is limited. However, many studies have reported that phytochemicals enhance gut health by altering the gut microbiota. Phytochemicals with senolytic effects can mitigate intestinal cell aging and synergistically improve the intestinal health. EGCG and chlorogenic acid combination exerts beneficial effects on cellular aging and gut microbiome. EGCG suppresses gut inflammation by reducing the pro-inflammatory cytokine levels, restores the *Firmicutes/Bacteroidetes* ratio, and increases the abundance of beneficial bacterial families, such as *Lachnospiraceae*, *Muribaculaceae*, and *Rikenellaceae* [47]. Adipose and intestinal tissues, which are prone to cellular aging, benefit from chronic EGCG consumption, which significantly attenuates the expression of DNA damage markers, cell cycle inhibitors, and regulators of age-related secretory phenotypes [48]. Additionally, EGCG suppresses the growth of pathogenic bacteria and preserves microbial diversity, thereby offering potential anti-aging benefits by mitigating cellular aging and gut dysbiosis. In a mouse model of IBD, procyanidin C1 treatment exerts protective effect by preventing inflammation and degradation of mucus layer [49]. Procyanidin C1 notably increases the abundance of *Akkermansia muciniphila* which supports the reconstruction of the mucus layer [50] Moreover, fecal transplants from mouse treated with procyanidin C1 demonstrated protective effects against IBD, suggesting that senolytic treatments modify the gut microbiota and its metabolites, which, in turn, exert anti-inflammatory effects on intestinal cells. In a Parkinson’s disease (PD) mouse model, oral administration of fisetin significantly increased the abundance of *Lachnospiraceae*, while reducing the presence of pathogenic bacteria such as *Escherichia–Shigella* and *Bacillus* [51]. *Lachnospiraceae* species produce butyrate, a beneficial short-chain fatty acid that reduces intestinal inflammation by inhibiting nuclear factor-κB activation [52]. Modifying the composition and diversity of gut microbiota may exert neuroprotective effects against neurodegeneration, suggesting a potential new treatment for PD. Although this study did not directly examine the senolytic effects of the phytochemicals, it indicates that these benefits of senolytics may be due to the selective removal of senescent cells.

Gut microbiota and intestinal epithelial lining are the primary sites of interaction between senolytics and the host immune system, influencing both intestinal immune homeostasis and permeability. Senolytic treatment mainly aims to inhibit age-related pathologies in the gut and its microbiota. Impacts of senolytic phytochemicals on gut microbiota are summarized in Table 2. Further investigation on the effects of senolytic drugs on short-chain fatty acids and microbial metabolism is crucial for a comprehensive understanding of these agents. A bidirectional relationship between the gut microbiome and cellular aging has been suggested. Metabolites secreted by the gut microbiome, obtained via the biotransformation of dietary components, directly affect the aging of intestinal cells. Conversely, the accumulation of senescent cells in the gut epithelium and fibroblasts, along with SASP, alters the intestinal cell function and immune activation [53]. These interactions highlight the complex and dynamic interplay among the gut microbiota, senolytics, and aging markers, necessitating further research to elucidate the underlying mechanisms.

## Conclusion

Although existing research on senolytic phytochemicals and their effects on the intestinal microbial environment during aging is scarce and lacks comprehensive evidence, previous studies have demonstrated their potential to improve the gut health in aging populations. Interactions between the intestinal wall and microorganisms become increasingly important with age, and the accumulation of senescent cells in the intestinal wall destabilizes the gut environment, contributing to systemic inflammation. Therefore, senolytic phytochemicals may enhance the gut microbial environment during aging. By targeting and reducing the burden on senescent cells, these compounds restore stability and reduce inflammation, thereby promoting a healthy gut microbiome. However, further research is necessary to validate their specific effects and elucidate their mechanisms of action.

## Figures and Tables

**Table 1:** List of senolytic phytochemicals.

Phytochemicals	Sources	Targets	Models	Ref
Quercetin (combination with Dasatinib)	Apples, onions, berries, and citrus fruits	PI3K/AKT ↓ Bcl-xL ↓	HUVECs and MEFs	[13]
Fisetin	Strawberries, apples, persimmons, onions, and cucumbers	PI3K/AKT ↓ Bcl-2 ↓	HUVECs and Ercc1^-/-^ MEFs	[16, 30]
Curcumin analog EF24	Turmeric	Bcl-2 ↓	WI-38, IMR-90, HUVEC, HREC	[35]
EGCG	Green tea	PI3K/AKT ↓ Bcl-2 ↓	3T3-L1	[36]
Procyanidin C1	Grape seed extract, unripe apples, and cinnamon	Cleaved caspase-3 ↑ ROS ↑ Mitochondrial p53 ↑ Cytochrome c release ↑ Membrane potential ↓	PSC27, WI38, HUVECs, MSCs	[37]
Gingerenone A	Ginger	Bcl-xL ↓ Cleaved caspase-3 ↑	WI-38	[38]
Oleuropein	Olive leaves and fruits	Cx43 ↓	Synoviocytes	[39]

PI3K, phosphoinositide 3-kinases; AKT, protein kinase B; Bcl-xL, B-cell lymphoma-extra large; Blc-2, B-cell lymphoma 2; ROS, reactive oxygen species; Cx43, connexin 43; EGCG, epigallocatechin gallate

**Table 2:** Impacts of senolytic phytochemicals on gut microbiota.

Phytochemicals	Model	Impacts on gut microbiota	Ref
Quercetin (combination with Dasatinib)	Aging (Mouse)	*Akkermansia muciniphila* ↑ *Firmicutes/Bacteroides* ↓ *Coprococcus* ↑ *Dorea* ↑ *Oscillospira* ↑ *Ruminococcus* ↑ *Sutterella* ↑	[45]
Fisetin	IBD (Mouse)	*Akkermansia muciniphila* ↑ *Firmicuties* ↑ *Verrucomicrobia* ↑ *Ruminococcus* ↑ *Proteobacteria* ↑ *Desulfovibrionaceae* ↑ *Deferribacteraceae* ↑	[46]
	PD (Mouse)	*Lachnospiraceae* ↑ *Escherichia-Shigella* ↓ *Bacillus* ↓	[51]
EGCG	Aging (Mouse)	*Lachnospiraceae* ↑ *Muribaculaceae* ↑ *Rikenellaceae* ↑ *Lactobacillaceae* ↓ *Erysipelotrichaceae* ↓ *Deferribacteraceae* ↓	[47]
Procyanidin C1	IBD (Mouse)	*Akkermansia muciniphila* ↑ *Helicobacter typhoons* ↓ *Sutterellaceae* ↑ *Verrucomicrobiaceae* ↑ *Erysipelotrichaceae* ↑ *Rikenllaceae* ↑ *Prevotellaceae* ↑	[49]

IBD, Inflammatory Bowel Disease; PD, Parkinson’s Disease; EGCG, Epigallocatechin gallate
